# Picky ABCG5/G8 and promiscuous ABCG2 ‐ a tale of fatty diets and drug toxicity

**DOI:** 10.1002/1873-3468.13938

**Published:** 2020-10-14

**Authors:** Narakorn Khunweeraphong, James Mitchell‐White, Dániel Szöllősi, Toka Hussein, Karl Kuchler, Ian D. Kerr, Thomas Stockner, Jyh‐Yeuan Lee

**Affiliations:** ^1^ Max Perutz Labs Vienna Campus Vienna Biocenter Center for Medical Biochemistry Medical University of Vienna Vienna Austria; ^2^ CCRI‐St. Anna Children’s Cancer Research Institute Vienna Austria; ^3^ School of Life Sciences Queen's Medical Centre University of Nottingham Nottingham UK; ^4^ Center for Physiology and Pharmacology Institute of Pharmacology Medical University of Vienna Vienna Austria; ^5^ Department of Biochemistry, Microbiology and Immunology Faculty of Medicine University of Ottawa Ottawa ON Canada

**Keywords:** ABCG2, ABCG5, ABCG8, ATP‐binding cassette, cholesterol efflux, membranes, multidrug resistance, polar relay, structural biology

## Abstract

Structural data on ABCG5/G8 and ABCG2 reveal a unique molecular architecture for subfamily G ATP‐binding cassette (ABCG) transporters and disclose putative substrate‐binding sites. ABCG5/G8 and ABCG2 appear to use several unique structural motifs to execute transport, including the triple helical bundles, the membrane‐embedded polar relay, the re‐entry helices, and a hydrophobic valve. Interestingly, ABCG2 shows extreme substrate promiscuity, whereas ABCG5/G8 transports only sterol molecules. ABCG2 structures suggest a large internal cavity, serving as a binding region for substrates and inhibitors, while mutational and pharmacological analyses support the notion of multiple binding sites. By contrast, ABCG5/G8 shows a collapsed cavity of insufficient size to hold substrates. Indeed, mutational analyses indicate a sterol‐binding site at the hydrophobic interface between the transporter and the lipid bilayer. In this review, we highlight key differences and similarities between ABCG2 and ABCG5/G8 structures. We further discuss the relevance of distinct and shared structural features in the context of their physiological functions. Finally, we elaborate on how ABCG2 and ABCG5/G8 could pave the way for studies on other ABCG transporters.

## Abbreviations

**ABC**, ATP‐binding cassette

**ECL**, extracellular loop

**ER**, endoplasmic reticulum

**ICL**, intracellular loop

**NBD**, nucleotide‐binding domain

**NBS**, nucleotide‐binding site

**TMD**, transmembrane domain

**TMH**, transmembrane helix

The ATP‐binding cassette (ABC) transporter superfamily is one of the largest families of membrane proteins in all living kingdoms. ABC transporters mediate substrate translocation across membranes, primarily using ATP hydrolysis to drive transport against concentration gradients [1, 2]. The vast majority of eukaryotic ABC transporters are exporters, although ABCA4 [3], yeast Pdr11, and Aus1 may act as lipid importers [4, 5]. Human ABC transporters are classified into seven subfamilies from ABCA to ABCG based on sequence similarity and domain organization [6]. A eukaryotic ABC transporter consists of at least four functional units: two highly conserved nucleotide‐binding domains (NBDs) and two transmembrane domains (TMDs). These four domains are typically expressed as a single protein, usually with domains arranged TMD‐NBD‐TMD‐NBD. Alternative arrangements such as a homo‐ or heterodimer of ‘half transporters’, each with one TMD and one NBD, usually arranged TMD‐NBD [7, 8]also exist [9].

The ABCG family of eukaryotic ABC transporters has several remarkable features that are unique within the wider ABC superfamily. Members of the ABCG family, including the five representatives in humans, are ‘reverse’ half transporters, with domains arranged in a NBD‐TMD configuration only found in the ABCG family [8, 10]. Notably, the closest orthologues of ABCG proteins are the yeast pleiotropic drug resistance (PDR) transporters, which share the reverse topology [11, 12]. Interestingly, four out of the five human ABCG transporters are involved in lipid transport [13]. Recent structural data have greatly enhanced our understanding about substrate specificity and transport mechanism in three human ABCG members. As structural information for ABCG1 or ABCG4 is unavailable as yet, we will focus this review on ABCG2, ABCG5, and ABCG8, but also discuss how this could lead to a better understanding of all ABCG members.

### Discovery and biology of ABCG5 and ABCG8

ABCG5 and ABCG8, originally referred to as sterolin‐1 and‐2, were first identified in patients with the rare genetic disease sitosterolemia, a disorder of sterol absorption and secretion that elevates plant sterol levels in plasma and tissues [14, 15, 16, 17, 18, 19, 20, 21]. The *ABCG5* and *ABCG8* genes are co‐expressed from a common promoter located on chromosome 2p21 at the *STSL* (sitosterolemia) locus in a head‐to‐head orientation [22, 23, 24, 25].

ABCG5 and ABCG8 are complexed as obligate heterodimers (ABCG5/G8) and predominantly expressed at the apical membrane of enterocytes of brush border villi on the small intestine, gall bladder epithelial cells, and canalicular membranes of hepatocytes. Both subunits are synthesized and assembled in the endoplasmic reticulum (ER), followed by N‐linked glycosylation and transport to the apical cell surface [26, 27, 28]. The post‐Golgi trafficking of the mature ABCG5/G8 may be regulated by sterols, bile acids, and cAMP signaling [29]. However, when each of them was expressed alone, the unstable half transporter proteins were retained in the ER in a calnexin‐dependent manner and degraded by proteasomes. Thus, co‐expression and folding of both halves is required for a functional ABCG5/G8 heterodimer [30].

Metabolic studies and animal knockout models confirm that liver‐resident ABCG5/G8 is required for efficient sterol excretion into the bile, with as much as 30‐fold increase in plasma accumulation of phytosterols such as sitosterol in ABCG5/G8 null mice [19, 26, 27, 28, 30, 31, 32, 33, 34]. In the small intestines, ABCG5/G8 limits absorption of sterols, and is now known as the primary sterol transporter of the transintestinal cholesterol efflux (TICE), accounting for 35% of the fecal cholesterol elimination. Hence, ABCG5/G8 provides an essential means to eliminate toxic sterols, thus reducing the risk of cardiovascular diseases [29, 35, 36, 37]. Of note, ABCG5/G8 is required for liver X receptor‐mediated induction of macrophage‐specific reverse cholesterol transport pathway that shuttles tissue‐generated high‐density lipoprotein particles back to the liver [38].

Sequence analysis of ABCG5 and ABCG8, particularly the ‘classical’ NBD catalytic motifs Walker A, B and signature sequences, fueled the notion that ABCG5 and ABCG8 play asymmetric roles in controlling cholesterol secretion *in vivo*. This idea was substantiated by observations showing the effects of mutations in either ABCG5 or ABCG8 [39, 40]. The precise mechanism remains elusive, but of note, bile acids increase basal ATPase activity of ABCG5/G8 and specifically promote cholesterol efflux in cell models [41, 42]. The recent exciting advances in determining the ABCG5/G8 crystal structure allow now for integration of experimental and mutational data with a molecular understanding of ABCG5/G8 transport function.

### Discovery and biology of ABCG2

Human ABCG2 was discovered in an adriamycin/doxorubicin‐selected breast cancer cell line, MCF‐7/AdVp3000 (MCF‐7/AdrVp), and originally named Breast Cancer Resistance Protein more than 22 years ago [43, 44]. Simultaneously, ABCG2 was mapped on chromosome 4q22 and highly expressed in placenta or mitoxantrone‐resistant cells, thus named the human placental ABC protein (ABCP) or mitoxantrone resistance [43, 44, 45, 46, 47]. ABCG2 was immediately a new focal point of interest in multidrug resistance (MDR) to a wide range of cytotoxic compounds independent of P‐glycoprotein (Pgp/ABCB1) [48] and MDR‐related protein (MRP1/ABCC1) [49].

ABCG2 shows a broad distribution in many cells and tissues, including at the plasma membrane of hematopoietic stem cells and erythrocytes, mammary alveolar cells, gastrointestinal tract epithelial cells, kidney proximal tubules, seminiferous cells of the testes and hair follicle stem cells, the blood–brain and blood–testis barriers, as well as in placental trophoblasts. Follow‐up studies quickly demonstrated an extremely broad ABCG2 substrate specificity, sharing both overlaps and distinct substrate sets when compared to P‐gp and MRP [50, 51]. Moreover, the broad ABCG2 substrate range contrasts with the sterol‐limited substrate selectivity of other human ABCG transporters (see below: Substrate Recognition and Selectivity in ABCG5/G8 and ABCG2). The ever‐growing list of substrates as well as inhibitors (often with some blurring of the distinction) today counts more than 200 compounds, including cancer and noncancer therapeutics, common dietary xenobiotics and environmental toxins [52, 53], and metabolites as well as vitamins [54].

Following its original isolation from a MDR cell line, deregulated ABCG2 overexpression significantly correlated with a pronounced MDR phenotype, along with poor prognosis and low survival in acute myeloid leukemia [55, 56, 57, 58, 59, 60, 61, 62, 63, 64, 65]. Studies in solid tumors also correlate ABCG2 overexpression with poor prognosis, including supporting evidence in small cell and non‐small cell lung carcinoma, though there is conflicting evidence for breast cancer [66, 67, 68, 69].

ABCG2 harbors several dozen single nucleotide polymorphisms (SNP) within the coding region [70], initially lacking a clear genotype–phenotype connection. However, genome‐wide association studies provided the first evidence for a physiological role of ABCG2 when the most frequent *ABCG2* polymorphism (C421A or rs2231142, leading to a Q141K replacement) turned out to be the most strongly predictive allele for hyperuricemia/gout [71]. Multiple subsequent studies confirmed this genetic association, and biochemical data now provide compelling evidence that ABCG2 is a physiologically important urate transporter, contributing to the nonrenal clearance of excess purine [72, 73, 74, 75].

A second physiologically relevant function for ABCG2 appears to be the transport of hemoglobin proto‐porphyrin metabolites such as pheophorbide A. Indeed, a light‐induced porphyria phenotype present in knockout mice confirms metabolite transport. Intriguingly, there are no reports of light‐induced pathologies of individuals with the junior blood group, which typically shows a loss of ABCG2 surface expression in erythrocytes [76]. Of note, animal models of erythropoietic protoporphyria suggest a role for ABCG2 in cytoprotective porphyrin transport [77], mirroring a likely protective role for ABCG2 as a physiological porphyrin transporter in hematopoietic stem cells [78]. With its broad substrate specificity, other definitive physiologically relevant substrates and/or metabolites of ABCG2 remain to be identified. However, it seems reasonable to state that ABCG2 is a pivotal and intrinsic guardian of tissues and organs in preventing cellular stress from unwanted substrates. ABCG2 is known to mediate physiological detoxification across most epithelial barriers ranging from placenta, gastrointestinal tract, and brain, even including the somewhat controversial amyloid peptide transport across the blood–brain barrier [54, 79, 80, 81, 82].

### Structure and mechanism of action of ABCGs in the low‐resolution era

Despite an initial low‐resolution EM structure for ABCG2 [83], our detailed knowledge of the structure and mechanism of ABCG family transporters came from mutational studies. Such studies revealed critical roles for various residues in ABCG2, G5, and G8 domains. For example, the cysteine residues C603 in the putative third extracellular loop 3 (ECL3) of each ABCG2 monomer form a disulfide bond, covalently linking and stabilizing the ABCG2 homodimer [84, 85]. Interestingly, the heterodimeric ABCG5/G8 lacks a stabilizing disulfide bond. The observation that ABCG1 and ABCG4 may form heterodimers, though not with ABCG2 [86], suggests that disulfide bridges may stabilize homodimers, by preventing incorrect transporter assembly when multiple ABCG proteins are simultaneously translated. Another key residue identified in ABCG2 was R482 in the putative transmembrane helix 3 (TMH3). Remarkably, R482 mutations markedly alter drug selectivity; replacing arginine by a negative charge expands the spectrum of recognized drugs, adding rhodamine 123 and doxorubicin to the list of ABCG2 substrates [87, 88, 89, 90]. Nonetheless, the overall knowledge about the mechanism and catalytic cycle of ABCG family members remained rudimentary (Box 1). Many important questions could not be addressed before the appearance of the first structure from the ABCG family: how would the reverse topology of the G‐family manifest itself in its structure? What are the functional or mechanistic implications of sequences unique to the ABCG family, including the large intracellular domain connecting the NBD with TMH1 or the large extracellular domain between TMH5 and TMH6? How can single residues such as R482 in ABCG2 control substrate selectivity, and could that be explained by high‐resolution structures?

**Available structural information on ABCG transporters before the crystallographic studies on ABCG5/G8.**

Structural information on NBD: Structural features known prior to the publication of the first ABCG5/G8 structure were mainly derived from the sequences of NBDs, which are highly conserved across the entire ABC superfamily. Hence, it was reasonable to assume that ABCG proteins would share the fold of NBDs, as confirmed by the atomic x‐ray structure of ABCG5/G8. In addition, biochemical data confirmed that conserved motifs have similar functions, thus supporting predictions from sequence comparisons, strongly suggesting a general NBD structure in ABC proteins.

Structural information on TMDs: The first attempt to determine a three‐dimensional structure of an ABCG transporter resulted in a low‐resolution 20 Å map of ABCG2 [91]. The maps displayed an inward‐facing conformation, with the NBDs widely separated from each other, and a drug‐transport path formed by TMDs. The authors also created a homology model of ABCG2 based on the ABCB‐type exporter Sav1866, assuming that ABCB and ABCG exporters share a similar fold. The model did not fit into the cryo‐EM density, thus providing the first structural hints for major differences between ABCB‐ and ABCG‐type exporters. Specifically, the overall height of ABCG transporters seemed shorter, while the extra density relative to the NBDs was later identified as elbow connecting helices.

Models of ABCG transporters: Comparative homology modeling is based on the assumption that proteins sharing sequence homology also share folds and thus structures. A known structure with a sequence identity higher than 20% can serve as a proper template structure [92]. Accordingly, the Sav1866 structure [93] with 28% identity served as a template for ABCG2 [94]. The generated models correctly predicted the fold of the NBDs, but due to NBD‐biased identity measure, comparative modeling was faulty, because valid templates for generating testable structural TMD models were unavailable. This highlights a limitation of comparative modeling, which requires that the fold is shared between the structural template and the target protein to be modeled.

Several ‘unified’ transport mechanisms for ABC exporters had been proposed [95, 96, 97, 98, 99]. For example, a general transport mechanism described that, upon ATP hydrolysis, alternating changes between inward and outward conformations of the TMDs provided the driving force for the transport cycle of ABC transporters [100]. This idea was consistent with the original alternating access model for membrane transporters, whose transport cycles should be accomplished by an alternative exchanges of protein conformations facing either extra‐ or intracellular compartments [101]. Here, accessibility to the substrate‐binding site alternates, but the binding site is never accessible from both sides of the bilayer. Further, attempts to unravel transport mechanisms by modeling ABCG transporter on ABCB family transporter coordinates were futile and quickly lost traction [10]. Hence, mechanistic similarities between diverse ABC members are likely restricted to the motion and ‘activation’ of NBDs [2] that determine the overall changes in the accessibility of substrate‐binding sites. A full understanding of ABCG2, G5, and G8 requires structural and atomic details regarding domain motions and dynamics, substrate pathways, and inter‐ as well as intradomain communication to allow for mechanistic conclusions.

## Structural fold and motifs of ABCG transporters

The X‐ray crystal structure of ABCG5/G8 and the cryo‐EM structures of ABCG2 were resolved in either inward‐facing or outward‐facing conformations [102, 103, 104, 105]. Both transporters share a similar fold that is unique among eukaryotic efflux transporters, yet they appear more similar to a subset of bacterial or mammalian uptake transporters [104], enabling model building for ABCA1 [106]. ABCG5/G8 and ABCG2 thus establish a paradigm for ABCG transporters, as well as for the ABC2 porter system [8, 107, 108].

### NBD

**Fig. 1:**
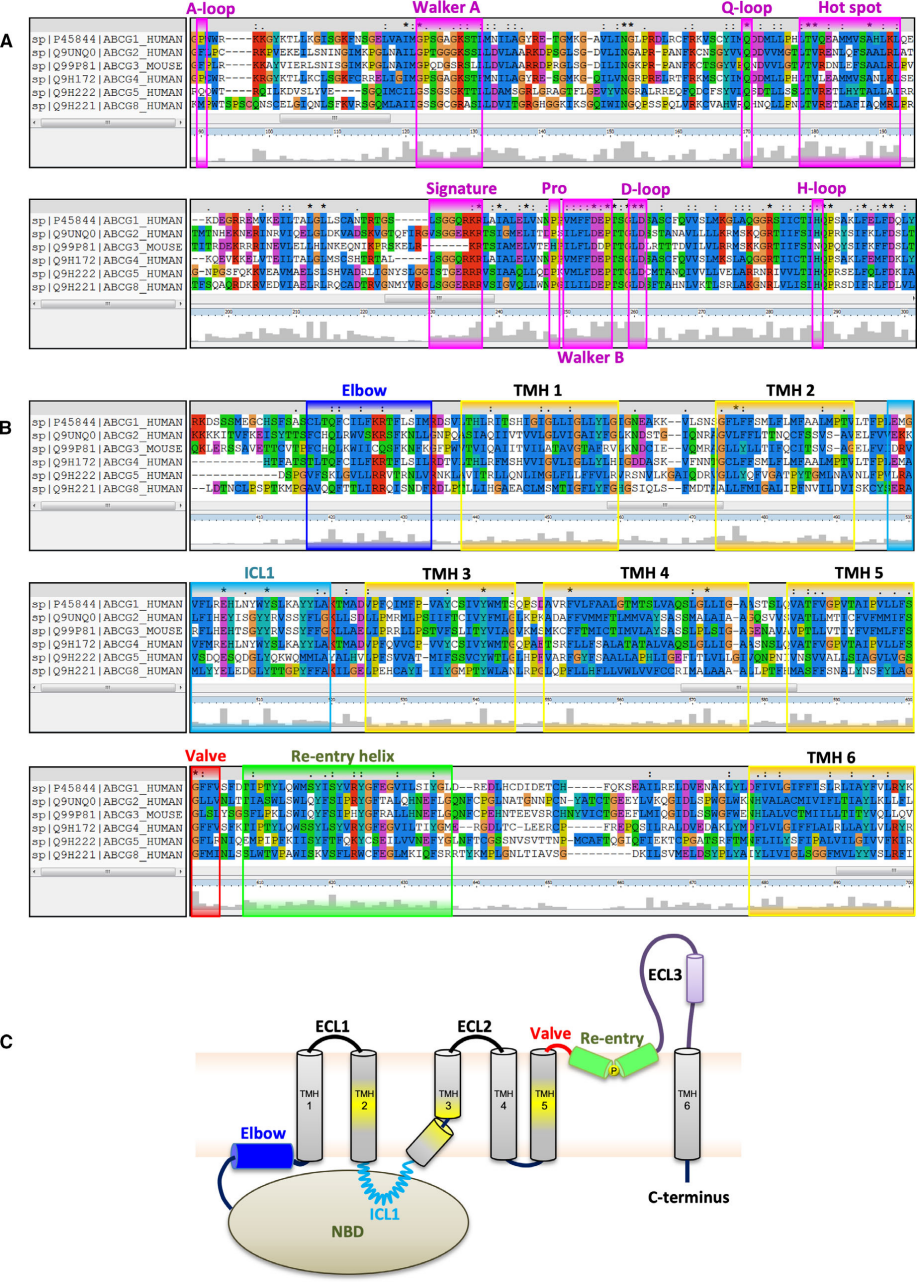
Sequence alignment of mammalian ABCGs. (A) MSA was analyzed using ClustalX2. Conserved residues are highlighted with the conservation scale as the height of gray bars at the bottom of each residue. Conserved regions in the NBD are highlighted in the pink boxes, including A‐loop, Walker A, Q‐loop, hot spot helix, Signature loop, Pro‐loop, Walker B, D‐loop, and H‐loop, respectively. (B) Conserved regions in the TMD. The elbow connecting helix (blue box), TMH1‐TMH6 (yellow boxes), ICL1 coupling helix (light blue box), leucine valve (red box), re‐entry helix (green box), respectively. (C) The putative topology model of mammalian ABCGs. Colors are as in panel B. The putative substrate binding is highlighted as yellow in TMH2, TMH3, and TMH5.

**Fig. 2:**
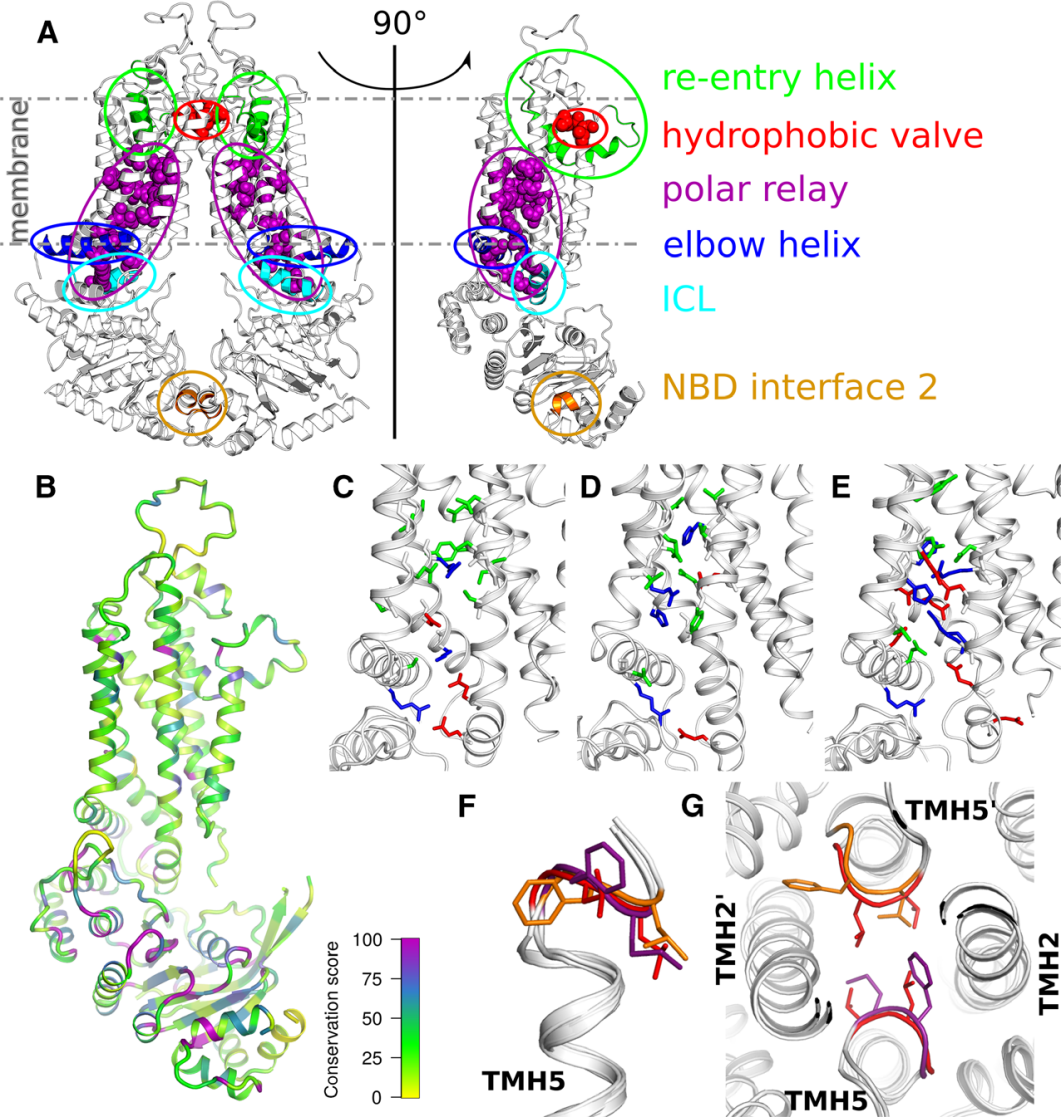
Common structural features and differences among ABCG2, ABCG5, and ABCG8. (A) Structural elements characteristic to the ABCG subfamily are indicated in ABCG2 (PDBID 5NJ3). The transporter is shown in side‐view as a functional dimer across the dimer interface as a monomer. (B) Sequence conservation among the human ABCG family members projected on the monomeric ABCG2 structure oriented as in panel A. Highly conserved regions are shown as thick purple tubes with a decreasing conservation toward yellow using the Clustal consensus score [112]. (C‐E) Zoom of the polar relay residues (acidic—red, basic—blue, polar—green) are shown as sticks on the monomeric cartoon representation of ABCG2 (C), ABCG5 (D), and ABCG8 (E). (F) Overlay of TMH5 and the immediately following loop that forms a hydrophobic valve across the transporter symmetry axes. Valve residues are shown as sticks and colored red for ABCG2, orange for ABCG5, and purple for ABCG8. (G) Overlay of ABCG2 and ABCG5/G8 fitted on TMH5, highlighting the hydrophobic valve residues from the extracellular side, color coded as in panel F.

The nucleotide‐binding sites (NBS) of either ABCG2 or ABCG5/G8 share a basic structure of the RecA‐like and the α‐helical subdomain, including all conserved motifs necessary for ATP binding and hydrolysis [109] (Fig. 1A). In ABCG5/G8, the NBD dimer is pseudosymmetric in the absence of nucleotides, whereas ABCG2 NBD homodimers are symmetric. At the intracellular end of the NBD, the two subunits form an intimate contact interface through a conserved NPXDF motif, named NBD interface 2 (Fig. 2A). Interestingly, the NBD dimer in ABCG2 remains always connected through this interface, thus providing structural stability during dynamic movements in the transport cycle [103, 104]. Of note, this cytoplasmic motif is conserved in all mammalian ABCG transporters and consistently, mutations in this interface in ABCG1 or ABCG2 also affect transporter functions, at least in cell models [110, 111]. The ATP‐bound ABCG2 shows nucleotides that are occluded between the two symmetric NBS, similar to other ABC transporters. In the absence of ATP, both ABCG2 and ABCG5/G8 open the NBS at about 35° angles to allow for nucleotide exchange [103, 104, 105].

### Triple helical bundle and transmission interface

The interface between the NBD and the TMD holds a ‘triple helical bundle’ formed by the elbow connecting helix, the first intracellular loop (ICL1) coupling helix and the ‘hot spot’ helix, also known as E‐helix (Fig. 2A). The elbow connecting helix forms an amphipathic helix with one side of membrane‐exposure (hydrophobic side), and the other one water‐exposed (hydrophilic side). This structure anchors and stabilizes transporters to the inner leaflet of the bilayer. The NBDs form a structurally conserved socket that interacts with the intracellular coupling helix from the TMDs, universally present in all ABC transporters [93, 102, 113, 114, 115]. In the ABCG family, ICL1 is the longest ICL1 that harbors the coupling helix at the transmission interface. This is in contrary with the other eukaryotic ABC folds as showed in ABCB/C/D structures, where the coupling helix typically resides in the last ICL. In addition, all ICLs of the ABCG family appear short when compared to those from the ABCB/C/D subfamilies. This thereby positions the NBD in close proximity to the inner membrane leaflet, similar to the ABCA family and the bacterial type II importers such as BtuCD [9]. All three helices show conserved stretches across the human ABCG family. The triple helical bundles may form the transmission interface during NBD‐TMD communication to facilitate the use of energy derived from ATP hydrolysis to trigger substrate translocation through the conformational switch [116]. Importantly, the conformations of ABCG2 suggest that the NBD motions couple through this triple helix transmission interface with the TMDs, leading to structural changes that open/close the substrate‐binding sites.

### TMD and polar relay

The membrane‐spanning domain of each monomer contains six transmembrane α‐helices (TMH1‐TMH6), with an N‐terminal elbow connecting helix and the large ECL3 that includes the re‐entry helix (Figs 1C and 2A). The ABCG5/G8 and ABCG2 structures lack a domain‐swapped topology as first observed in Sav1866 [93], but appear to share a side‐by‐side arrangement similar to type II bacterial importers [114]. The TMD‐TMD interface in ABCG5/G8 appears collapsed and sealed, while the dimerized TMD in ABCG2 reveals two apparent cavities. The central cavity is located within the membrane‐embedded TMHs, while a much smaller but visible upper cavity is present in the extracellular region. The cavities hold binding sites that are connected by a putative translocation pathway for substrate expulsion. Interestingly, the central cavity in the inward‐facing state is much larger, possibly explaining the broad specificity of ABCG2 [103, 104, 105]. Importantly, the transmembrane bundle harbors a region called the *polar relay*, which contains several conserved polar residues that are embedded in the core of each transmembrane region, connected through a series of hydrogen bonds and salt bridges. Mutational change of one conserved residue (Y432) in the polar relay of ABCG5 affects biliary cholesterol secretion in a mouse model [102], suggesting a critical role of the polar relay for ABCG5/G8 function. The ABCG2 R482G or R482T mutants in TMH3 alter substrate specificity, expanding the substrate overlap with ABCB1 [117]. Interestingly, the M523A and F640A mutations, which are on the same axis parallel with the membrane as R482, enhance transport activity [118]. TMH5, which resides in the translocation pathway, rotates 180° upon substrate binding, turning several sulfur‐containing side chains toward the central core in an the *apo* state [105]. Remarkably, cryo‐EM structures of apo‐ABCG2 in the presence and absence of the 5D3 antiboby show different architectures at TMH2, TMH5, and the substrate‐binding pocket [104, 105, 119], strongly suggesting that F_ab_ binding disturbs the overall fold and conformational dynamics of ABCG2 during catalytic cycle and drug transport. Hence, a cautious approach is required when solving and interpreting structures from particles that bind high‐affinity antibody fragments [103, 104].

### Extracellular membrane interface

The extracellular side is composed of several structural elements, including a hydrophobic valve, the re‐entry helix, and the ECLs, which cooperate to ensure substrate release (Figs 1C and 2A). Interestingly, each of these domains in ABCG2 contains at least one critical residue essential for ABCG2 function [120]. The re‐entry helices are highly conserved but only present in the ABCG family. In ABCG5/G8, the re‐entry helices and the transmembrane helices form a vestibule configuration at the lipid‐membrane interface, where cholesterol‐like electron densities are seen in the crystal structure [102]. The A540F mutation on TMH5 of ABCG5 prevents the sterol binding and abolishes the biliary secretion of cholesterol *in vivo*. This residue, localized just outside the hydrophobic valve, shares the TMD interface with ABCG8, and may thus be the main entry site for sterol recognition.

The hydrophobic valve in ABCG2, also known as the dileucine valve, forms a hydrophobic seal in the putative translocation channel to prevent water leakage between two cavities [120]. In ABCG2, E585 in the re‐entry helix forms a salt bridge with R426 in ECL1 to stabilize the conformation at the extracellular interface [120]. Superimposing outward‐ and inward‐facing structures demonstrate that both re‐entry helices show minimal motions between these two conformations. The ECL3 is the largest loop and contains several polar residues, forming a polar roof‐like structure covering the top of ABCG2. This architecture is further stabilized by the intramolecular disulfide bond of C592 to C608, and the intermolecular disulfide bond between C603 of each monomer [84, 85]. The ECL3 also contains N‐linked glycosylation sites, such as N596 in ABCG2 [121], N585 and N592 of ABCG5, and N619 of ABCG8 [30]. Of note, glycosylation is essential for 5D3 antibody recognition of ABCG2 [122, 123, 124, 125]. The 5D3 monoclonal antibody is a conformation‐sensitive antibody with extremely high affinity for ABCG2. Its binding restricts dynamic movement of ABCG2, especially of ECL3‐connected regions, thus inhibiting both ATPase and transport function [126].

### Structural difference between ABCG2 and ABCG5/G8

The structures of ABCG5/G8 and ABCG2 are highly similar. Hence, the ABCG5/G8 structural coordinates were essential for solving the structure of 5D3‐bound ABCG2 particles [103], as well as for structural modeling of ABCG2 [116, 127]. Multiple sequence alignment (MSA) of protein families can reveal conserved residues and domains likely to be important for function under different selection pressures. Some will be universally conserved, where all proteins conserve specific residues or the chemical property of residues, indicating this position is critical for the entire protein family. Others will be divergently conserved, where each subgroup in the MSA is conserved at a given position, although the position may differ between groups. The MSA of ABCGs from 29 mammalian species revealed many universally conserved as well as divergently conserved positions. Figure 2A highlights common features that differentiate ABCG transporters from ABCB, ABCC, or bacterial transporters. Closer inspection reveals differences in the NBDs, the polar relay, and the hydrophobic valve. Sequence comparison shows that the ABCG family NBDs have a higher conservation when compared to the TMDs, which may explain the distinct substrate spectra (Fig. 2B). While ABCG2 harbors consensus Walker A and signature motifs, this is not true for ABCG5 and ABCG8 [40]. ABCG5 holds a degenerate signature motif (ISTGE, instead of LSGGQ/E), whereas ABCG8 contains a degenerate Walker A (GSSGCGRAS, RA instead of KS/T) rendering one of the NBS inactive with respect to ATP hydrolysis. Introduction of the same mutations to the intact site yields an inactive transporter *in vivo*. Interestingly, some of the NBD mutations in ABCG5 abolish cholesterol, but not plant sterol transport, indicating a critical structural role for the degenerate NBD [39, 40]. It is tantalizing to speculate that the asymmetry of the NBDs is somehow transmitted to TMDs, resulting in the observed variation in substrate selectivity.

The polar relay [102] is a highly charged structural element sandwiched in between the TMD regions. The relay stretches from the cytosolic membrane aspect to the center of the lipid bilayer. These polar and charged residues from TMH1‐TMH4 are located within the transporter core. Such high polarity within the core is very unusual for a membrane protein, but the residues are not membrane‐exposed, and only a few are on the surface and in contact with water. Such polar interactions might increase structural flexibility when compared to a hydrophobic core and thus provide a flexible hinge. The relay is highly conserved in the ABCG subfamily (Fig. 2B‐E), but astonishingly, the residue identity shows an unexpectedly high variability, whereby ABCG8 contains the highest number of charged residues and ABCG2 the lowest. Some of the polar relay residues are at the dimer interface and the substrate‐binding site, suggesting a role in maintaining TMD dimers as well as in substrate binding. Salt bridge‐forming residues at the elbow connecting helix–TMD interface in ABCG2 [116] are crucial both for folding and transport, although ABCG8 lacks an equivalent salt bridge.

**Fig. 3:**
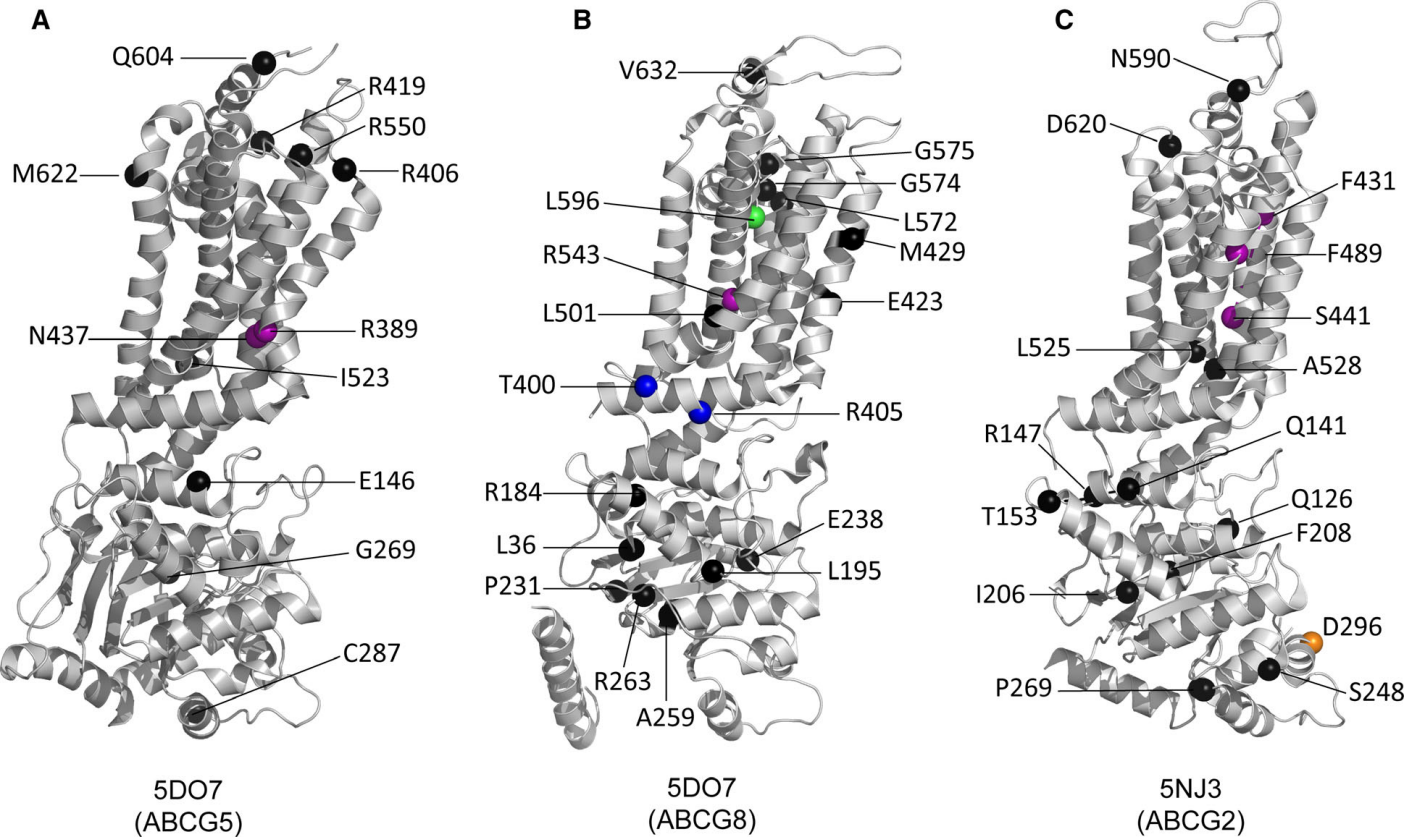
Localization of disease‐causing mutations and SNPs in ABCG2, ABCG5, and ABCG8. The positions of disorder‐related polymorphisms or mutations are highlighted as the colored spheres on the structures of ABCG5 (PDBID 5D07, chain A), ABCG8 (PDBID 5D07, chain B), and ABCG2 (PDBID 5NJ3). The color code is based on the structural motifs as shown in Fig. 2; NBD interface (orange sphere), elbow connecting helix (blue spheres), polar relay (purple spheres), and re‐entry helix (green sphere); otherwise, other residues are in black sphere, respectively. All details are indicated in Table 1.

The double leucine motif in ABCG2 (L554, L555) is important for function [120], but its sequence is only partially conserved. The two leucine residues are phenylalanines in most other mammalian ABCG transporters, suggesting that a GΦΦ motif forms the gate in the translocation channel between the substrate‐binding cavity and the upper ligand release cavity [120]. A comparison of this gate in known ABCG proteins shows that they share a common loop structure (Fig. 2F,G) directly following TMH5. Further, position and orientation of the hydrophobic residues leucine and phenylalanine is shared. They appear symmetrically oriented around the central C2 rotational axis, thereby sealing the putative substrate translocation path. A comparison between the inward‐facing and the outward‐facing ABCG2 conformations indicates that this transition changes the interactions in the dileucine motif residues across the dimer interface, suggesting a valve‐like mechanism for opening a path during substrate translocation [120]. Interestingly enough, mapping residues that can appear in SNP variants, most disease‐causing missense mutations reside within or near the triple helical bundles, the TMD polar relay, the re‐entry helices, or the hydrophobic valves (Fig. 3 and Table 1). Hence, these novel structural motifs and the configuration conserved between ABCG5/G8 and ABCG2 are likely to play essential roles in regulating transporter functions, and may therefore hold therapeutic promises for pharmacological targeting of pathological SNPs.

**Table 1:** Disorder‐causing mutations in ABCG5, ABCG8, and ABCG2.

Location	Motif	Gene	Mutant	Related disorder(s)	References
NBDs	NBD hot spot	ABCG5	E146Q	Sitosterolemia	[128]
		ABCG2	Q141K	polymorphism, gout, CML, NSCLC, MCC, GIST, AD	[71, 75, 129, 130, 131, 132]
			R147W	polymorphisms	[133]
	NBDs	ABCG5	R50C^a^	non‐Sitosterolemia	[134]
			G269R	Sitosterolemia	[135, 136]
			C287R	Sitosterolemia	[137, 138]
		ABCG8	D19H^a^	non‐Sitosterolemia	[139]
			Q24H^a^	Sitosterolemia	[140]
			L36P	polymorphisms	[141]
			Y54C/H^a^	non‐Sitosterolemia	[139, 142]
			R184H	Sitosterolemia	[34]
			L195Q	Sitosterolemia	[128]
			P231T	Sitosterolemia	[34]
			E238L/K	Sitosterolemia	[34, 143]
			A259V	Sitosterolemia	[34]
			R263Q	Sitosterolemia	[34]
			E340Q^a^	polymorphisms	[134]
		ABCG2	V12M^a^	polymorphism, gout, breast cancer	[125, 144, 145, 146, 147]
			Q126X	polymorphism, gout	[72, 148, 149]
			T153M	polymorphisms	[125, 150, 151]
			I206L	polymorphisms	[123, 151]
			F208S	polymorphism, gout, CML	[152, 153]
			S248P	polymorphisms	[124, 154, 155]
			P269S	polymorphisms	[130, 156, 157]
			D296H	polymorphism, CML	[153]
TMDs	Extracellular membrane interface	ABCG5	R406Q	Sitosterolemia	[136]
			R419P/H	Sitosterolemia	[19]
			R550S	Sitosterolemia	[34, 128])
			M622V	polymorphisms	[139]
		ABCG8	L572P	Sitosterolemia	[34]
			G574E/R	Sitosterolemia	[34]
			G575R	polymorphisms	[34]
	Inner membrane leaflet	ABCG5	I523V	polymorphisms	[139]
		ABCG2	L525R	polymorphism, CML, NSCLC	[153, 158]
			A528T	polymorphisms	[153]
	TMD polar relay	ABCG5	R389H	Sitosterolemia	[159]
			N437K	Sitosterolemia	[139]
		ABCG8	R543S	Sitosterolemia	[34]
		ABCG2	F431L	polymorphisms	[124, 151, 154, 155, 158, 160]
			S441N	polymorphisms	[124, 154, 156, 158, 161]
			F489L	polymorphisms	[124, 154, 155, 158]
	ECL3	ABCG5	C600Y^a^	polymorphisms	[139]
			Q604E/K	polymorphisms, non‐Sitosterolemia	[19, 139]
		ABCG8	V632A	non‐Sitosterolemia	[142, 162]
		ABCG2	N590Y	polymorphisms	[123, 151, 163]
			D620N	polymorphisms	[122, 123, 124, 125, 153]
	Elbow connecting helix	ABCG8	T400K	polymorphism, non‐Sitosterolemia	[139]
			R405H	Sitosterolemia	[34]
	Re‐entry helix	ABCG8	L596R	Sitosterolemia	[34, 139]
	TMDs	ABCG8	E423D	Sitosterolemia	[164]
			M429V	polymorphism	[138]
			L501P	Sitosterolemia	[34]
			Y641F	Sitosterolemia	[139]
			L650R	polymorphism	[165]

^a^ Unresolved on structural models.

## NBD‐TMD crosstalk and substrate translocation pathway

### Regulation of catalytic symmetry at the NBD

In ABCG5/G8, NBS1 is adjacent to the triple helical bundle of ABCG5, where two arginine‐glutamate pairs, R374‐E452 and R381/R377‐E146, stabilize the helical bundle and form a rigid body. On the other hand, NBS2 is near the triple helical bundle of ABCG8, where one arginine–glutamate pair shows no interaction, and possibly allows for flexible conformational changes during signal transmission at the active site [102]. Because the helical bundles are located at the NBD‐TMD interface, their direct interaction with the NBS supports the notion that they contribute to the flexibility and catalytic specificity at the asymmetric ATP‐binding sites in ABCG5/G8 [39, 40] as well as the symmetric NBDs in ABCG2 (N. K. Khunweeraphong and K. K. Kuchler, unpublished data). By contrast, ABCG2 requires two tightly packed monomers to form a symmetric and fully functional transporter, with the apo‐structure opening up the sites for ATP binding as well as substrate entry. The catalytic cycle may be initiated by the binding of two ATP molecules at the NBS. ATP acts as a molecular glue that connects the NBDs into a nucleotide sandwich dimer, thus generating an occluded state to trap ATPs and substrates inside the transporter [109]. NBD closure initiates, contributes to or triggers the conformational changes which communicate with the TMDs through the triple helical bundle (NBD‐elbow‐ICL1 cluster) at the transmission interface (N. K. Khunweeraphong and K. K. Kuchler, unpublished data). The shared interface of both NBDs at the bottom is critical for ATPase activity [111]. ATP hydrolysis then triggers the NBD dissociation and resets the machine into an inward‐facing state ready for another catalytic cycle. Of note, cholesterol enhances substrate‐stimulated ATPase activity and the catalytic cycle of ABCG2 from *Sf9* cell membranes [166, 167]. Cholesterol interaction is also evident in the cryo‐EM structures of ABCG2, with one residing at the substrate‐binding pocket. Thus, membrane cholesterol may exert a regulatory role for ABCG2 function [166, 167, 168].

In addition, despite being a symmetric nondegenerate dimer, some evidence suggests that ATPase activity of ABCG2 is uncoupled and independent from substrate transport [116]. Some substrates do not stimulate ABCG2 ATPase activity, a property shared with yeast Pdr5, suggesting a similar mechanism [169]. Of note, yeast PDRs such as the full‐length transporter Pdr5 contain at least one degenerate NBD, yet both ABCG2 and Pdr5 can act as uncoupled transporters [169]. One aspect of this enigmatic catalytic cycle is whether a single consensus NBD‐binding site is necessary and sufficient to drive conformational changes and transport function. Such uneven catalytic cycle and presence of at least three drug interaction sites predict that one ATP is always bound to an NBD to stimulate the binding of the second ATP molecule. In addition, hydrolysis of one ATP is sufficient to drive transport by ABCG2 [118]. Although the NBDs share highly conserved sequences and folding, the coupling mechanisms in the catalytic cycles for exporters and importers might be entirely different or at least subject to distinct regulation [170]. Speculatively, the functional consequence of such uneven catalytic cycle may be that the ABCG2 homodimer has two ATP‐binding sites but that they are always in different states, similar to the ATP‐binding sites of the bacterial F‐ATPase [171]. Thereby, they are structurally identical but functionally asymmetric. Such catalytic asymmetry may be solidified in the case of heterodimeric ABCG5/G8 during the evolution.

### Regulation at transmission interface for NBD‐TMD communication and substrate access

**Fig. 4:**
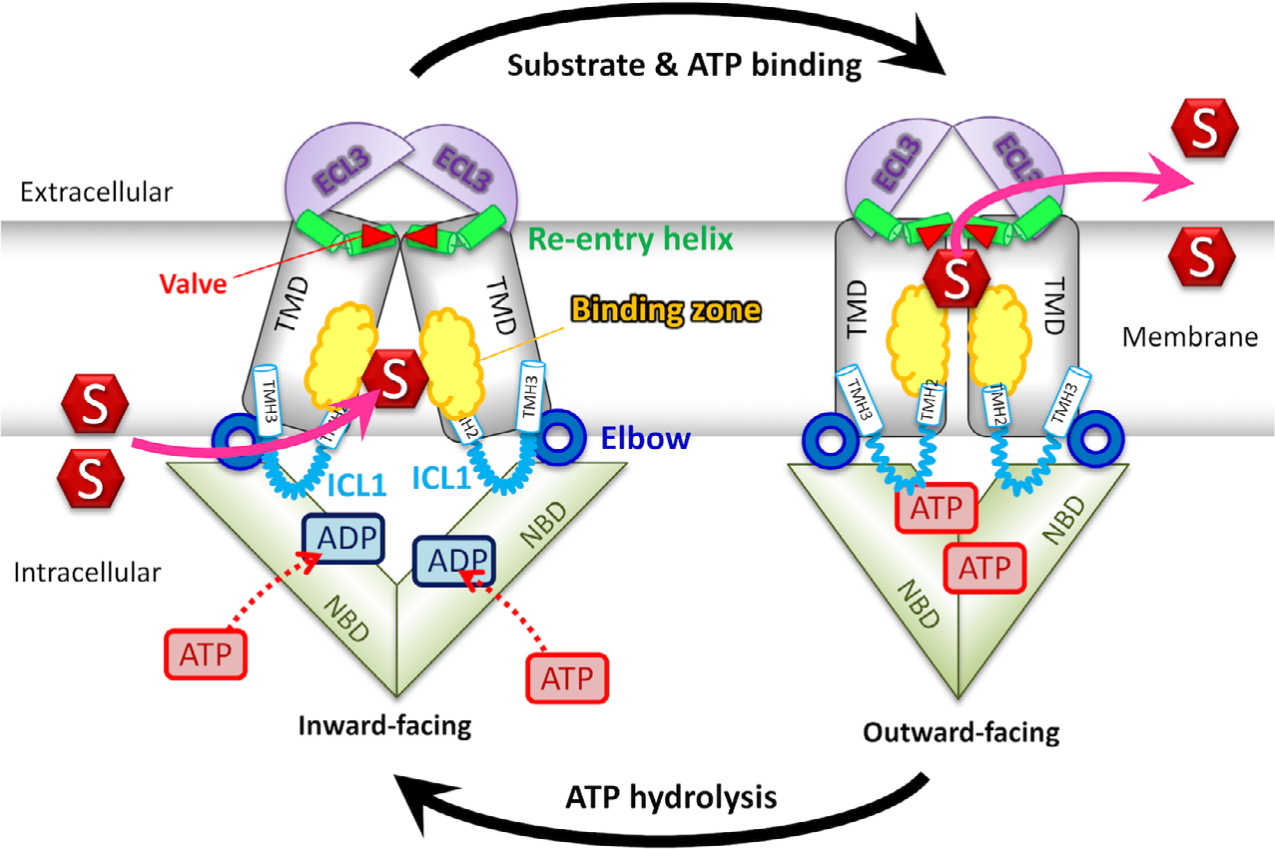
Proposed transport mechanism. ABCG transporters rest in the inward‐facing state. Substrate selectivity is determined by binding zone residues, which partially overlap with the polar relay in the core. Substrates (S) can enter either from the cytoplasm or from the inner leaflet of the lipid membrane. Substrate recognition may trigger ATP and substrate binding, followed by a transporter switch to the outward‐facing conformation. Substrate is extruded either into the extracellular medium or to the membrane outer leaflet. ATP hydrolysis resets the transporter to the inward‐facing state ready to bind either ATP or substrates if available.

The movements during NBD dimerization induce dynamic changes at the transmission interface. The transmission interface of the inward‐facing state of ABCG2 is more open and provides enough space for substrate entry into the translocation pathway. The elbow connecting helix restricts the flexibility of the NBD‐elbow‐ICL1 cluster during the catalytic cycle and functions as a hinge‐like rotational axis. ICL1 plays a key role as a coupling helix for the NBD‐TMD communication and operates as a molecular spring [116]. Glutamate 451 (E451) is essential for ABCG2 function and localizes to the core of the triple helical bundle. E451 may be involved in promoting drug entry by acting as or controlling a intracellular gate at the transmission interface allowing for substrate access (Fig. 4) [116, 172, 173]. Moreover, the NBD dimerization compresses the central cavity, which is believed to generate peristaltic pressure as a driving force to push substrates toward the top of the central cavity but also to aid the conformational switch [119, 120].

### Regulation at the central cavity and polar relay for substrate recognition

The TMDs of ABCG2 contain the substrate/inhibitor recognition sites. They also surround and create the putative substrate translocation pathway. Glutamate 446 (E446) in TMH2 turns its side chain into the middle of the central cavity, which is crucial for transport function and substrate recognition. E446 may also contribute to forming the substrate‐binding pocket [116, 137]. For example, mitoxantrone reaches the ABCG2 binding pocket after lateral ‘trapping’ from the lipid bilayer, followed by expulsion across the plasma membrane [2, 174]. Phenotypes of alanine mutations in residues next to R482 and in TMH3 support the notion of a ‘surface‐binding’ site and a buried site serving in mitoxantrone efflux [118]. These binding sites may communicate in a continuum and in a highly dynamic manner. In addition, the lipid‐exposed F640 in TMH6 of ABCG2 faces the intracellular region at the TMH2‐TMH3 interface and might be involved in drug access [118], which is consistent with M523A and F640A mutations enhancing transport activity.

Molecular dynamics (MD) simulations of ABCG5/G8 predict motions [102], showing that the elbow connecting helix and ICL1 of both ABCG5 and ABCG8 move inwards each other, which brings the triple helical bundles in closer proximity. This inward movement is coupled with an upward movement of the conserved polar residues along the polar relay, which may be disrupted by the sitosterolemia mutation R543S in ABCG8 [102, 175]. Using coevolution analysis, long‐range interactions allowed for predicting four pairs of conserved residues, whereby each pair is more than 8 Å apart in the apo‐structure [102, 175]. Finally, the shape and space of a putative ABCG2 substrate translocation channel is determined and controlled by dynamic movements of TMH2 and TMH5 [120]. The conserved F439 in the middle of TMH2 is a critical residue for substrate and inhibitor recognition, since it holds aromatic side chains from both monomers as clamps to trap substrates or inhibitors in the binding pocket [104, 105, 119].

## Substrate recognition and selectivity in ABCG5/G8 and ABCG2

### Regulation of substrate transport at the extracellular membrane interface

ABCG5/G8 features two pseudosymmetric vestibules at the protein–membrane interface, possibly extending into the TMD region. The X‐ray data suggest cholesterol‐shaped densities near these vestibules, and accordingly, may be the sterol‐binding sites [102]. This notion of sterol binding is supported by the A540F mutation in ABCG5, since this change inhibited biliary cholesterol excretion. The core TMD interface, the re‐entry helix, and the ECL harbor disease‐causing mutations, similar to analogous substrate‐exit mutations in the *Drosophila melanogaster* white gene [175, 176]. Together, the TMD vestibules may act in concert, providing the entry portal and exit gateway of sterols from the membrane bilayer. In ABCG2, at least two key features at the extracellular interface regulate drug release. First, the dileucine valve (L554/L555) controls drug movement from the central cavity to the upper cavity. Furthermore, it seals the valve to regulate substrate translocation and prevent water leakage between the cavities. Second, a polar roof acts as an extracellular gate to regulate access to the upper cavity, accepting substrate before release into the extracellular side. The roof forms a compact but flexible lid‐like structure. In the outward‐facing state, the volume of the upper cavity becomes enlarged by ~ 3‐fold when compared to the inward‐facing state. This change holds substrates with low binding affinity and ensures efficient drug extrusion, while at the same time preventing their re‐uptake [120]. Indeed, studies from other ABC exporters imply a similar function for ECLs acting as the extracellular gate to prevent substrate rebinding after release or re‐uptake [173, 177, 178].

### Single and multiple substrate‐binding sites

The burning question concerning the number and location of substrate/inhibitor binding sites in ABCG2 remains controversial and heavily discussed even among authors of this review. The atomic structures of ABCG2 suggest a first common site in cavity 1 [104]; however, pharmacological data argue that multiple drugs can bind the transporter though it remains unclear how different chemical species access the same central binding site [118]. Hence, other cryptic drug‐binding sites may exist that operate in a highly dynamic and transient manner. Mutational studies, biochemical experiments, and a conformation‐sensitive antibody such as 5D3 decorating the inward‐open conformation [126, 179] suggest several residues that can affect ABCG2‐mediated substrate transport [88, 89, 90, 104, 116, 118, 180, 181]. All in all, the data strongly suggest that substrate specificity is affected by the TMD conformation and by dynamic changes in helix packing that shape substrate‐binding regions and thus substrate affinity. Conformation‐perturbing mutations may alter protein dynamics without a need to be in direct contact with the substrate, explaining the many mutations affecting transport function, as well as ATPase activities.

ABCG2 substrates often differ in both size and lipophilicity, and it is reasonable to speculate that some compounds bind to a wider open TMD inward‐facing conformation than others. Hence, modifications, such as the catalytically inactive E211Q mutant, will exclude only a group of ligands and affect specificity and affinity. Consistent with this, some mutations will only alter substrate‐inducible ATPase activity [89, 104, 116]. Indeed, some structures show that residues affecting substrate transport are often not in direct contact with substrates and located far from suspected binding sites. Moreover, a single mutant may affect transport of a distinct substrate set [87, 111, 118], demonstrating that both substrate selectivity and transport activity are affected by mutations that affect overall conformational dynamics. It remains elusive whether these effects reflect multiple binding sites in ABCG2, complex or cooperative binding sites, kinetics of interactions [4, 118, 182] or allosteric modulation of transporter architecture. Indeed, cooperative binding of daunomycin and negative cooperativity between daunomycin and doxorubicin binding in the R482G mutant [90] supports allosteric modes of action. Further, this provides strong evidence for compounds which can bind simultaneously or consecutively, and structural data provide examples of either one or two ligands binding at the same time (Table 2). Larger ligands seem to bind across the symmetry axis of the ABCG2 dimer, occupying both symmetry‐related binding regions, and preventing the binding of a second ligand [104, 105].

**Table 2:** Structures of ABCG2 with bound ligand or substrates.

PDBID	Ligand name	Ligand count	Reference
6ETI	BWQ or MZ29	2	[104]
6FFC	BWQ or MZ29	2	[104]
6HIJ	BWQ	2	[104]
6VXH	imatinib	1	[105]
6VXI	mitoxantrone	1	[105]
6VXJ	SN38	1	[105]
6HCO	estrone 3‐sulfate	1	[104]
6FEQ	MB136	1	[104]

When discussing substrate‐binding sites or zones, substrate entry needs to be considered as well. Due to the strict symmetry of the ABCG2 dimer, entry may proceed through two symmetry‐related access paths. The entry sites are also open to the water–lipid interface, therefore providing access for substrates from within the lipid bilayer or from the cytosol. After binding to the transporter, two scenarios are feasible. First, inhibitors remain bound owing to a high affinity, high on‐rate interaction that fixes the transporter in the bound conformation by impairing conformational flexibility. Second, substrates have lower affinity recognition and fall ‘victim’ to the ATP‐binding driven conformational dynamics that evicts substrates. Hence, ‘good’ substrates will eventually leave the transporter through the dileucine valve, reaching the upper cavity, while the transporter proceeds through the transport cycle. Inhibitors would remain bound to a transporter, suggesting that the main difference between inhibitors and substrates would be their affinity, the kinetics of interaction [4] with the recognition sites which is also determined by their the lipophilicity.

As for ABCG5/G8, the pronounced sterol selectivity may in fact support this notion, with cholesterol acting as bound modulator/inhibitor of function and sitosterol being a preferred substrate. It is also tempting to speculate that the binding sites are actually general compound binding ‘zones’, and substrate specificity is mainly set by a ‘filtering’ process while substrates are moving through the entry gate or through the translocation channel [127] or in the region of the sterol density present in the ABCG5/G8 structure. A gate as access filter would be in line with altered TMD conformations leading to a change in the substrate spectrum such as seen for the R482G mutant. The mere fact that ABCG2 can interact with more than 200 compounds, and the size of the central cavity, strongly supports the existence of binding zones, instead of a well‐defined binding site. A single mutation could still abolish the entire transport, as it could cause long‐range effects on distant regions in the binding zones or impair conformational dynamics changing the volume and shape the substrate‐binding zone.

### Evolutionary considerations affecting function

Sequence alignment reveals conserved residues and domains likely to be important for function under different selection pressures (Figs 1 and 2B). These conservation patterns imply that at least some functional differences within the ABCG family arise from differences in transmission between domains. For instance, the hot spot helix is the most conserved within the triple helical bundle at the transmission interface, speculatively playing a crucial role in NBD‐TMD crosstalk during the catalytic cycle. All conserved residues in this region share their interfaces as a conserved network of NBD‐elbow‐ICL1, such that any mutational changes in this helix affect mature protein stability (N. K. Khunweeraphong and K. K. Kuchler, unpublished data). Such notion was supported by the presence of a very common variant Q141K of ABCG2, indicating an important regulatory helix at the transmission interface [71, 75, 127, 129]. There is also a preponderance of positions uniquely conserved in ABCG5 at the protein:lipid interface toward the extracellular face. Interestingly, this region also holds a high number of positions uniquely conserved in ABCG1 and in ABCG4 though to a lesser degree. The lid‐like extracellular roof is important for the release of substrates by ABCG2 [120]. Another important interface for ABCG2 is the L554 and L555 hydrophobic valve separating the central and the upper cavities (Figs 1B and 2G). The aliphatic L554 is however replaced by phenylalanine in the vast majority of other ABCG sequences (136 out of 141) (J. Mitchell‐White & I. D. Kerr, unpublished). Many residues are conserved in other combinations of ABCG family members, but these patterns in functionally separable groups suggest that at least some differences in substrate specificity result from dynamic communication between transporter domains rather than the promiscuity of binding sites.

## Conclusion and Perspectives

### Do ABCG2 and ABCG5/G8 share a conserved transport mechanism?

While atomic structures of ABCG2 and ABCG5/G8 are essentially superimposable, ABCG2 and ABCG5/G8 dramatically differ in subunit composition, symmetry, and substrate specificity. First, heterodimeric ABCG5/G8 shows notable asymmetry between the two subunits, and there are differences in the conserved residues along the polar relays. ABCG2 holds a central dileucine valve at the extracellular membrane interface, whereas in ABCG5/G8 the hydrophobic residues are phenylalanines. The functional implications of this residue difference are unclear, but it is reasonable to speculate that the larger aromatic phenylalanine is critical for sterol selectivity. Interestingly, mutations to smaller residues in ABCG2 compromise transport function, while larger residues partially retain function [120]. Importantly, symmetric ABCG2 has two canonical ATP‐binding sites, ABCG5/G8 has only one, which is competent for a high enough ATP hydrolysis sufficient to support substrate transport. Secondly, a wealth of literature documents a broad spectrum of substrates for ABCG2. By sharp contrast, ABCG5/G8 shows a highly restricted substrate selectivity confined to cholesterol and phytosterols, although both transporters share many common structural features (Fig. 2).

Notably, they both show a closed cytoplasmic NBD interface 2 (at the intracellular end of the transporter). It seems plausible that this region may play a role in sensing cytoplasmic signals for transporter functions, similar to a regulatory domain in the maltose transporter [183]. Both transporters show an NBD‐TMD interface as part of an intimate network of triple helical bundles, with the latter including the hot spot or E‐helix, elbow connecting helix and the ICL1 coupling helix. Both transporters are fueled by ATP hydrolysis through NBD motions to drive the catalytic cycle, including a conformational switch that sweeps *via* these triple helical bundles on through the TMDs. The polar relay provides structural stability, but also allows for changing helical packing and perhaps rotation of the TMDs, thereby exploiting ATP hydrolysis for TMD bending.

The third ECLs engage the re‐entry helices to anchor the extracellular roof on top of the outer lipid leaflet and to enable dynamic motions of the top end of TMDs. This forms a shield for the hydrophobic valve and protects the path of substrate translocation inside the membrane. Remarkably, disease‐causing mutations in ABCG5/G8 are present in these regions [175]. Likewise, ABCG2 holds conserved residues of pivotal importance near this structural motif [118, 158]. These similarities thus suggest that both transporters share the importance of re‐entry helices for the mechanism of substrate transport. Furthermore, it is tempting to propose that the enigmatic and distinct spectra of substrate selectivity between ABCG2 and ABCG5/G8 may be buried in the extracellular domains, the valve and the roof.

Even subtle structural differences in the conformational dynamics of both transporters may explain different functions, together with specific contributions of evolutionary amino acid substitutions. For instance, the ABCG5/G8 structure holds a much smaller, narrower substrate‐binding cavity between the TMD subunits when compared with ABCG2. This difference in cavity sizes may be due to unique features of solving structures by single‐particle analysis versus crystallography or the presence or absence of substrates such as sterols during freezing or crystal formation. In addition, the hydrophobic valve in ABCG2 is formed by a defined dileucine motif that separates two cavities, while the valve in ABCG5/G8 appears open, perhaps owing to the larger the aromatic side chain of phenylalanine. Instead of a substrate‐gating role as proposed in ABCG2, the hydrophobic valve in ABCG5/G8 may simply allow transit or capturing of sterol substrates from the lipid bilayer during the conformational change at the TMD.

### Outlook

#### Challenges to reveal all conformations of the full transport cycle

Structural analyses of ABCG2 and ABCG5/G8 agree on numerous biochemical and genetic data, and these in turn confirmed the validity of the atomic structures. Importantly, these structures can spark additional biochemical and biophysical approaches to complete the mechanistic view of structure–function relationships and to decipher ABC transporter cycles. Currently, only a handful of crystal or cryo‐EM structures are available. Several structures for inward‐facing and/or drug/inhibitor‐bound conformations [103, 104, 105] exist, yet there is only one for a nucleotide‐bound occluded outward‐facing structure [104]. Thus, there is an urgent need for more atomic structures, ideally corresponding to intermediate conformations adopted during the transport cycles. It goes without saying that this will remain a major challenge in the coming decades, but the increase in achievable resolutions for membrane proteins by either cryo‐EM or X‐ray holds great promise.

Most importantly, to be able to assemble all structural data into full mechanistic views of catalytic cycles, there is still an unmet need for close cooperation between structural biologists and biochemists. A lack of such cooperation in the past has led to wrong interpretations of structural data and even to wrong structures. There is also a need to develop new tools to validate whether or not a given atomic structure, even if obtained at high resolution, is actually reflecting a biologically relevant conformation rather than a crystallizable energy minimum.

Importantly, structures are static snapshots in the path of the full transport cycle, but conformational changes are dynamic and thus difficult to capture for relevant states of the transport cycle, including nucleotide‐bound and/or substrate‐bound or release states. A major challenge for structure determination lies in protein sample preparation of membrane proteins, including the stabilization of the transporter molecules after solubilization, restoring proper and, as much as possible, native lipid environments to maintain functional states for ATPase or the transport cycles. The necessary knowledge will only come from rigorous biochemical, biophysical, pharmacological, and computational characterizations of the transporters. This will have to address the pivotal dynamic dimensions and establish the mechanistic sequence of events along the path of the transport cycle. As for the ABCG family, the exact functions of domains such as re‐entry helices or NBD interface 2 remain to be determined, and establishing their molecular and cellular roles will be essential to dissect and pin down the transport cycles.

#### Role of the membrane component to transporter functions

The natural environment for ABCG5/G8 and ABCG2 is the lipid bilayers of the plasma membrane with hundreds of different types of lipids, including cholesterol‐rich regions and areas of pronounced lipid asymmetry. The dominant lipid components of mammalian plasma membranes are sterol, phospholipids, plasmalogens, and sphingolipids. These membranes are not just passive permeation and communication barriers, as it has long been recognized that they also have an important signaling function utilizing arachidonic acid‐derived prostaglandins or leukotrienes, all of which are substrates for ABCC family members [49], as well as phosphatidylinositol 4,5‐bisphosphate (PIP_2_) or the PIP_2_‐derived inositol‐3‐phosphate and the diacylglycerol phosphatidic acid pathways. In addition to signaling, the ABCG family and ABC transporters at large demonstrate the critical roles that membrane lipids play for structural, functional, and regulatory aspects [184]. Early on it was recognized that membrane protein functions are sensitive to detergent purification and reconstitution. In recent years, differential functionality of several ABC transporters has been observed in either phospholipid bilayers or detergent‐phospholipid micelles, such as BtuCD [185], MsbA [186], MRPs [49], or Pgp [187], demonstrating the essential roles of lipid components for ABC transporter function. Indeed, cholesterol is essential for ABCG2‐mediated drug efflux [166], and ABCG5/G8 is physiologically expressed on the cell surfaces, where it contributes to resistance against high concentrations of bile acids [42]. The physiological substrates of ABCG5/G8 are cholesterol, plant sterols, and other small steroid molecules, consistent with the observation that ABCG5/G8 dysfunction causes sitosterolemia [18, 36, 128].

It would come as no surprise to see how changes in membrane lipid and/or protein composition can affect the activities of transporters from the ABCG subfamily. In fact, differences in tissue‐ or cell‐specific lipid environments could explain distinct transport functions. To support this speculation will require the functional reconstitution of native and purified ABCG family members in their *native* membrane lipid environments. Unfortunately, the impact of the membrane lipids on ABC transporter functions remains understudied. With the advancement in membrane protein reconstitution [188, 189] and MD simulation of large macromolecules, the existing structures and models of ABCG5/G8 and ABCG2 should promote interdisciplinary biochemical, biophysical, and computational approaches to elucidate the impact cellular membrane environments on ABC transporter function.

## Author contributions

NK, JMW, DS, and TH drafted the manuscript and generated figures and tables. KK, IDK, TS, and JYL edited the manuscript. The final version was approved by all authors. NK, JMW, DS, and TH contributed equally.
